# Support for All in the UK Work Programme? Differential Payments, Same Old Problem

**DOI:** 10.1111/spol.12058

**Published:** 2014-03-06

**Authors:** James Rees, Adam Whitworth, Elle Carter

**Affiliations:** a Third Sector Research Centre, University of BirminghamBirmingham, UK; b Department of Geography, University of SheffieldSheffield, UK

**Keywords:** Welfare-to-work, Employment services, Creaming and parking, Conditionality, Work Programme, Payment by results

## Abstract

The UK has been a high profile policy innovator in welfare-to-work provision which has led in the Coalition government's Work Programme to a fully outsourced, ‘black box’ model with payments based overwhelmingly on job outcome results. A perennial fear in such programmes is providers' incentives to ‘cream’ and ‘park’ claimants, and the Department for Work and Pensions has sought to mitigate such provider behaviours through Work Programme design, particularly via the use of claimant groups and differential pricing. In this article, we draw on a qualitative study of providers in the programme alongside quantitative analysis of published performance data to explore evidence around creaming and parking. The combination of the quantitative and qualitative evidence suggest that creaming and parking are widespread, seem systematically embedded within the Work Programme, and are driven by a combination of intense cost-pressures and extremely ambitious performance targets alongside overly diverse claimant groups and inadequately calibrated differentiated payment levels.

## Introduction

In common with much of the advanced economies (Lødemel and Trickey 2001), since the arrival of New Labour in 1997 the UK has made a decisive shift from a ‘passive’ to an ‘active’ welfare system in which eligibility for out of work benefits is tied increasingly tightly and explicitly to the stated obligation to seek paid work. This policy shift has been justified philosophically through the reciprocal relationship between rights and responsibilities embedded within the Third Way (Blair and Schroder 1998; Giddens 2000; Powell 2000) and has resulted in considerable development of, and evolution in, ‘activating’ welfare-to-work programmes throughout the 2000s. With the implementation of the Work Programme from 2011 the UK has come closer to joining the ranks of other advanced economies in embracing outsourced provision, devolved decision-making around intervention design and payment by results.

This evolution in recent decades in welfare-to-work services across the advanced economies is a result of several interrelated trends and concerns. There have been significant shifts in the organization of services with the implementation of contracting out and the creation of quasi-markets to respond to the critiques of public choice theorists around claims of unresponsive ‘bureaucratic’ state institutions and, more recently, the desire to transfer risk away from government (Considine 2000; Bredgaard and Larsen 2008; Mythen *et al*. 2012). These organizational reforms have been closely associated with the turns towards new managerialim and contractualism as well as shifts to ‘new’ governance modes involving changed relationships between the state, citizens and disadvantaged groups (Ramia and Carney 2000; Considine 2001; Struyven and Steurs 2005). Lastly, the influence of paternalism (Mead 1986) on policy discourse, framing and making has strengthened consistently in the UK since the late 1990s such that the Third Way balance between rights and responsibilities at the level of the individual has shifted to place greater onus on individual obligations. Moreover, at policy level there has been a parallel shift in emphasis away from the ‘carrots’ of policy supports (e.g. childcare, ‘making work pay’) toward the ‘sticks’ of sanctions-backed conditionality in response to alleged behavioural ‘defects’ (cf. DWP 2010a). Taken together, the aim for policymakers has been to create more efficient and specialized provision that is also more flexible, responsive and personalized to the different needs of unemployed individuals, and against which the unemployed have limited options but to participate on the terms set out to them.

As discussed in detail below, the evolution of welfare-to-work programmes in the UK through to the current Work Programme is, in a variety of ways, clearly influenced by these trends. Of particular interest to the present article is the extent to which the specific design of the Work Programme is able to realize the key tenets underpinning international welfare-to-work reform over the past two decades – efficiency, effectiveness, personalization, value-for-money, innovation, flexibility – within a programme design which appears to create multiple tensions and vulnerabilities around achieving these objectives. Of particular focus here is the challenge for the Work Programme to calibrate the incentives for providers to work differently in terms of meeting specific support needs – what Lister (2003) might term a process of ‘differentiated universalism’. In contrast to this aim, the international literature has consistently raised fears that in such outsourced, payment by results welfare-to-work schemes (particularly private) providers would respond to financial pressures and incentives by ‘creaming’ off easier to serve claimants whilst ‘parking’ harder to service clients (Struyven and Steurs 2005; Considine *et al*. 2011). These fears have been strongly aired in the UK context from the earliest days of the Work Programme (PAC 2012) and have escalated, both as the economic backdrop has become more challenging than anticipated during the design phase and as evidence has accumulated during the early months of the scheme expressing concerns of creaming and parking (Newton *et al*. 2012; Lane *et al*. 2013; PAC 2012, 2013; WPSC 2011, 2013; Rees *et al*. 2013). The argument from the Department for Work and Pension (DWP) is that the specific design of the Work Programme – the existence of minimum service guarantees and, in particular, the use of nine claimant groups with differential payments across each – would effectively mitigate incentives for prime providers to cream and park, and the public statements of the DWP remain consistent in arguing that there is no evidence to suggest that this is not working (PAC 2013). Within this context, the present article draws on analysis of recently published official Work Programme data alongside qualitative research on providers' experiences (Rees *et al*. 2013) in order to explore early evidence around creaming and parking in relation to the structures and incentives of the Work Programme's design.

The remainder of the article proceeds as follows. In the following section the evolution of welfare-to-work policies in the UK since the late 1990s is outlined with an emphasis on drawing out the key principles and aims underpinning that evolution. This is followed by a summary of the international evidence on creaming and parking, its risks for the Work Programme in light of the scheme's stated aim of delivering ‘differentiated universalism’ (Lister 2003) in welfare-to-work support and the ways in which the detail of the scheme's design seeks to mitigate those risks to achieve that aim. The quantitative and qualitative material is then used to analyze the evidence and experiences around creaming and parking while the last section discusses the findings and reflects on the implications for Work Programme design and for international learning.

## The Path to the Work Programme: A Radical Departure from an Established Trend

On coming to power in 1997 after almost two decades in opposition, New Labour quickly embarked on a series of ‘activating’ New Deal welfare-to-work schemes. Various New Deals were created for different claimant groups with different levels of conditionality. New Deal for Young People, for example, was a mandatory scheme which famously offered ‘no fifth option’ for those refusing to participate whilst those considered to have more ‘legitimate’ reasons for not working – most notably disabled adults and single parents – were offered voluntary schemes. In common across all of the New Deals, however, was a clear emphasis on supply-side measures and a ‘work first’ strategy of propelling working-age welfare recipients back into the labour market as swiftly as possible (Peck and Theodore 2000). In shifting the policy focus clearly to the supply-side, demand-side issues around weak local labour markets and job availability were largely rejected as ‘old Left’ and not feasible in today's global economy (Blair and Schroder 1998). Unemployment was, therefore, recast largely as an individual problem of employability rather than a structural problem of insufficient employment availability in local areas, a shift which has been the focus of considerable critique (Theodore 2007; Wright 2011) but which is seen particularly strongly and explicitly in the influential Freud report (Freud 2007). Supplementing these New Deals were a raft of policies to ‘make work pay’, most notably the introduction of the national minimum wage, tax credits and benefit run-ons. These incentivizing policies were combined with greater attention than previously to the framework of policy supports needed, particularly improving the availability of affordable childcare through the UK's first ever National Childcare Strategy and associated policies, such as childcare subsidies for low-income workers, Sure Start and free childcare places for three and four year olds.

If the early New Labour years were marked by the rapid construction of this activating welfare-to-work architecture then much of the following decade can perhaps best be described as one of ‘creeping conditionality’ during which there was a consistent trend of ratcheting up work-related behavioural requirements as well as the extension of these work-related conditionality requirements to traditionally inactive groups, particularly single parents and the disabled (Dwyer 2004). A critical juncture in the evolution of UK welfare-to-work policy was the publication of the Freud report in 2007 which received cross-party support and which was heralded as setting out the principles for welfare-to-work policies in the forthcoming decade: outsourcing and competition; ‘black box’ delivery models in which the state allows providers complete freedom over intervention design; personalized support; and payment by results. Arguing that unemployment is now frictional rather than structural, Freud also argued that enhanced conditionality and sanctions were needed to tackle the alleged behavioural causes of worklessness and the existence of a ‘dependency culture’, despite compelling evidence to the contrary (DWP 2010b; Shildrick *et al*. 2012). In the face of the empirical fragility of some of its key claims, the Freud report has, nevertheless, been highly influential to subsequent policy formulation. Almost immediately it set the template for New Labour's subsequent reformulation in 2009 of the main New Deal programmes into one Flexible New Deal (FND) in which welfare-to-work support was outsourced from Jobcentre Plus (the public sector employment support agency covering the UK) to external providers after one year of Jobcentre Plus support (or six months for fast-tracked claimants). In FND, financial payments to providers flowed mainly from successful job outcomes.

The arrival of the Conservative-Liberal Democrat Coalition government following the 2010 general election heralded a change in policy from the FND to the Coalition's Work Programme but also a continuation – indeed, a radical extension and intensification – of the principles set out in the Freud report (2007) and on which FND was designed. Although reflecting continuation from FND in terms of underlying principles the Work Programme, introduced in June 2011, is in various ways a genuine revolution in employment support policy, most notably in terms of the extent of sub-contracting and payments weighted to job outcomes as well as the ‘black box’ model of delivery. Delivery of the Work Programme takes place through contracts between the DWP and large-scale, mainly private sector prime providers which can both deliver services themselves and/or sub-contract to organizations within large and (sometimes) complex supply chains sitting underneath each prime. In very broad terms sub-contractors are either ‘tier 1’, delivering end-to-end services to participants throughout their time on the Work Programme, or ‘tier 2’, which contract with primes or tier 1s to provide specific interventions to participants. The Work Programme is structured geographically in the sense that Great Britain is divided into 18 large ‘regional’^1^ Contract Package Areas (CPAs) with two or three primes in each CPA to whom claimants are randomly allocated from Jobcentre Plus if they have not found work within an initial period of Jobcentre Plus provision, the duration of which depends largely on the type of out-of-work benefit received and the Work Programme claimant group in which they are therefore placed. Unlike FND, which contained mandatory service components, a ‘black box’ delivery model operates in the Work Programme so that providers have almost complete flexibility over their interventions, with only minimum service delivery guarantees (which are themselves of variable ambition, detail and potential enforceability) set out by each prime provider (Finn 2012). This flexibility is required given that, unlike the various group-specific New Deals, Work Programme has to cater for the needs of all different types of largely long-term unemployed claimants within a single employment scheme, in part reflecting planned changes to the benefits system in the form of the consolidation of most of the major benefits and tax credits within the single Universal Credit from 2013.

## Creaming, Parking and Differential Payments in the Work Programme

Creaming and parking by providers have long been considered endemic problems within welfare delivery systems involving outsourced provision combined with outcomes-based payments (Finn 2011), and international experience of similar welfare-to-work models highlights the extent of these issues in practice (Heckman *et al*. 2002; Dockery and Stromback 2001; van Berkel and van der Aa 2005; Finn 2011). ‘Creaming’ refers to providers skimming off clients who are closest to the labour market and targeting services on them in the expectation that they are more likely to trigger an outcome payment. ‘Parking’ refers to the opposite process, where those individuals deemed to be unlikely to generate an outcome payment are de-prioritized, perhaps receiving the minimum service specified in the contract. The issue is closely related to the more general economic literature around the difficulties in effectively managing principal-agent relationships via contracts (Bredgaard and Larsen 2008), and is made more likely where regulatory control or organizational norms or incentives against it are low – most obviously where providers are private organizations attracted to participation in welfare provision due to a simple profit motive. Of the 40 contracts won within the Work Programme, 35 were won by private sector primes, three by third sector organizations and two by public sector organizations, and fears of creaming and parking were strong from the outset. These concerns intensified in the context of tighter than expected cost-pressures on primes due to a combination of a more difficult than expected economic environment which affected job outcome (and hence payment) levels, the prevalence – and apparent success of – discounting practices at the bidding stage, and lower than expected caseloads within some payment groups (Inclusion 2011a).

International evidence suggests that creaming and parking by providers is widely experienced across different countries (Considine 2000; Struyven and Steurs 2005; van Berkel and van der Aa 2005; Bredgaard and Larsen 2008; van Berkel *et al*. 2012; de Graaf and Sirovatka 2012). The literature also makes clear, however, that the detail of programme design and payment structures can play a role in either mitigating or facilitating such provider behaviours (Considine 2000; Struyven and Steurs 2005; van Berkel and van der Aa 2005; Considine *et al*. 2011; Finn 2011, 2012). In bringing together such a diverse range of claimants into one single programme the DWP was aware from the outset that this was a challenge for the Work Programme and sought to mitigate these risks through the programme's design. This was attempted partly through the requirement for primes to set out minimum service guarantees but primarily through the placement of each individual into one of nine Work Programme claimant groups based on the type of benefit received as a proxy for the level of their perceived support needs. These claimant groups are important to providers because they carry with them different entry requirements and, crucially, different levels of financial reward for job outcome payments, scaled according to some notion of the average difficulty of transitions to employment for each claimant group (NAO 2012; Lane *et al*. 2013). To adopt Lister's terminology the issue is one of ‘differentiated universalism’ (Lister 2003) – seeking equality whilst (indeed, through) recognising difference – whereby policymakers seek to use the differentiated payments across claimant groups to incentivize Work Programme providers to treat different claimants *differently* dependent upon their distance to the labour market and barriers to work, in order that all claimants receive the amount and type of support so as to *equalize* opportunities to move into employment. Payments across these claimant groups vary from a maximum of £3,810 for Jobseekers' Allowance (JSA) claimants aged 18 to 25, to £13,720 potentially for an individual within the Employment Support Allowance (ESA) group for recent Incapacity Benefit (IB) claimants. As Robert Devereux, Permanent Secretary of the DWP, explained to the Committee of Public Accounts in February 2011, this is a step on from previous programme design in the UK in the field of welfare-to-work: ‘This set of prices, as has just been said, begins to move us towards trying to reflect some of the average difficulty … Everything we have done here takes us really a long way forward compared with where we were’ (PAC 2012).

The DWP's hope is that, if designed appropriately, differentiated payments across claimant groups would translate the policy rhetoric of differentiated universalism into policy reality, mitigating providers' incentives to cream and park. Compared to FND, however, and to most comparable international welfare-to-work schemes operating an outsourced payment-by-results financing model, the Work Programme weights a smaller share of the provider's potential payment to the initial attachment – or joining – fee and a far larger share to employment transitions and, in particular, sustained job outcomes (generally measured in the Work Programme as six months of sustained employment). Within FND the ratio between the initial joining fee, a successful transition into work and a sustained job outcome was roughly 40:30:30 (Vegeris *et al*. 2011:13) whilst in the Work Programme the ratio is closer to 10:25:65,^2^ although it varies somewhat across the nine claimant groups. With performance outcomes, therefore, mattering to a far greater degree in Work Programme than in comparable previous schemes it becomes critical to successfully mitigating the economically rational incentives to cream and park amongst primes both that the level of financial payments *between* payment groups realistically reflects the relative difficulty of moving claimants within these groups into (sustained) employment *and* that these claimant groups are relatively homogeneous *internally* such that a single level of payment realistically reflects the needs of all claimants within each claimant group. If either of these assumptions is not satisfied then there should be a logical expectation that creaming and parking will take place if providers are assumed to be economically rational and if they are confident that creaming and parking will go undetected and/or unpunished. In the following section the overarching question which the discussion of the quantitative and qualitative material seeks to answer is a simple one: does it appear that the DWP has succeeded in designing a scheme which mitigates against providers' incentives to cream and park?

## Data and Methods

The quantitative analysis draws on the Work Programme's official statistics published online by the DWP. These statistics show the numbers of unemployed people referred and attached to the Work Programme and numbers of job outcome payments made to providers as a result of participants achieving sustained employment (six months of employment or three months for members of ‘harder to help’ payment groups) (DWP 2013). We construct the DWP's preferred ‘job outcome rate’ measure (job outcomes/referrals) from the most recent official programme statistics, and this covers the period from programme launch in 2011 to the end of September 2013. The qualitative analysis draws on a research project which involved key informant interviews and case studies of delivery in two localities (Rees *et al*. 2013). The eight key informant interviews included respondents from third sector and employment services infrastructure organizations, private and third sector prime contractor organizations, and some large national third sector organizations delivering the Work Programme as sub-contractors. The case studies of delivery were located in two areas chosen to provide geographical and labour market diversity (inner-city vs. semi-rural, north vs. south England) and different supply chain models. In each area a brief ‘mapping’ exercise identified the role and type of organizations in the supply chains. This was followed by a phone survey of these sub-contractors (approximately 65 per cent contacted) which confirmed a number of their basic characteristics, their position and role in the supply chain and the nature of their provision. These issues were further explored in two focus groups with sub-contractors (one each for tier 1 and tier 2 providers) held in one of the localities. Lastly, interviews were conducted with four of the five private sector primes operating in the two sampled areas (the fifth declined to take part) and 14 of their sub-contracted providers.

## Differential Payments but Still Differential Outcomes: Rhetoric vs. Reality in the Work Programme

As a first step in understanding claimants' differing needs, profiling tools have become increasingly commonplace within welfare-to-work programmes both in the UK and internationally. Within the Work Programme prime providers are adopting a whole range of approaches to profiling and using these analyses to guide (at least in the first instance) the intensity and type of interventions targeted at the individual (Newton *et al*. 2012: 47–9). Tellingly, however, rather than adopting the DWP's claimant groups as the structure for their activities, prime providers tend to develop their own streams of claimants and related intervention packages, suggesting that the differentiated payments embedded within the Work Programme's claimant groups may well not correspond to providers' view of claimants' distance to the labour market. Rather, commenting on the providers' use of these profiling tools one provider suggested that the RAG (red, amber, green) rating system^3^ used by some primes and their end-to-end providers to ‘triage’ their caseloads was, in effect, a mechanism for creaming those rated ‘green’, focusing energies and resources on those easiest and most likely to move into work, whilst parking claimants rated ‘red’ who are considered to need more time and resource to support back into work. Asked if this was the case, one sub-contracted provider stated:

*‘That's done openly. [At the first customer assessment] you'd give an anticipated job start date and you categorise people on day one into red, amber and green categories … So from day one you're categorised and if you're a green customer you've got an anticipated job start date, you've got an action plan to work towards that, and you have to be seen so that is once or twice a week. So you're pushed. If you're amber your job start date is obviously further away, and it's the expectation that you'll have activity at least once a fortnight. If you're red it could be a phone call once a month. So people are not using the word parking because it's politically incorrect, but it's happening.’* (tier 2 provider)

Indeed, Work Programme providers with long-standing experience of welfare-to-work provision argued that such practices were not just endemic but that they could also be seen as a rational response to the current payment by results model and its misalignment with the actual support needs of individual claimants across and within the claimant groups. Nevertheless, while interviewees expressed the view that most providers would cream if given the chance there were a number of ways in which potential mechanisms for creaming could be shut off. One was strict random allocation of jobseekers between a prime's ‘in-house’ delivery and delivery by their ‘end-to-end’ delivery partners. One prime claimed this was preferable in any case because it permitted proper comparison of performance between providers in the supply chain and, therefore, improved performance management, but it was impossible to verify whether this system could in reality be circumvented. The incentive for primes to cream skim could be removed entirely where they outsourced all delivery to their sub-contractors, as is the case with a number of primes in the Work Programme; but certainly this may in effect push the issue down to the sub-contractor level. Whilst feeling that creaming and parking were hard to avoid in the current design given the intense dual pressures around costs and targets, not all providers were comfortable with these practices. This provider, for example, was uncomfortable behaving in this way towards claimants but felt torn by the need to deliver the targets for the organization within the budget available:

*‘So we are going to have these numbers of customers that perhaps may never find employment in the two years. We'll never be paid for them either but we'll be paid for the other 50% that are likely to go on into work so there's a level of parking going on which we're not particularly comfortable with but we also need to achieve what we need to achieve and what the primes need to achieve so it's trying to get a balance really.’* (tier 1 provider)

However, another provider argued that parking was a sensible way to manage the caseload and that the extent of parking would need to be assessed over the full length of the attachment period:

*‘I think from a provider perspective we are expected to prioritise customers that are coming through who are job ready and to move those through as quickly as we can, and I think from a financial perspective that's realistic because you've got to get the money in the system to keep it all flowing. I don't think you purposefully park people but it could seem like that from the outside because it's taking longer to get those people job ready or … they're being referred onto, say, drug and alcohol services who will be working with them and until they have their condition managed then you can't work with them. So there might be a perception of parking because it's taking longer and efforts are, at this group, to move them through [the system].’* (tier 2 provider)

**Figure 1:**
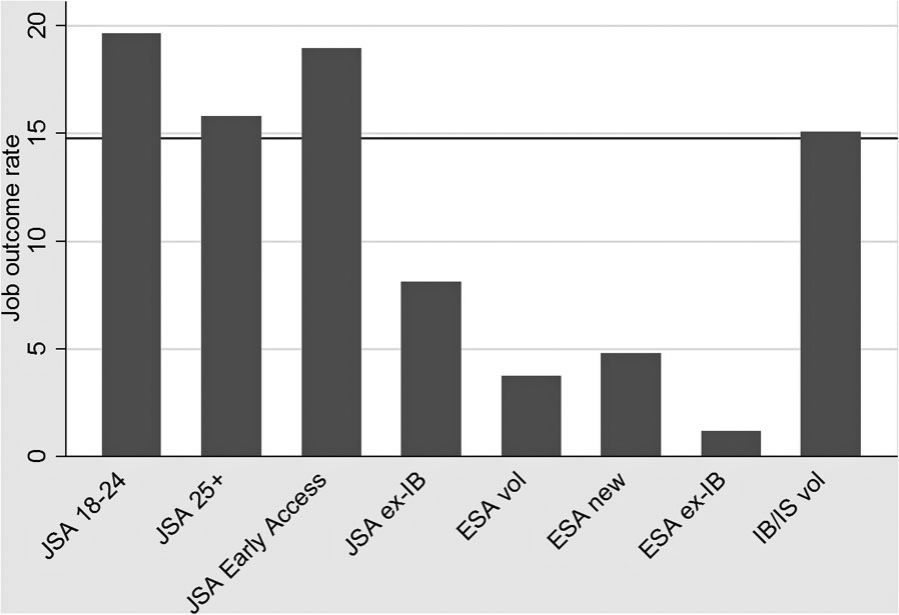
Differential job outcomes between Work Programme claimant groups

To try to get a sense of the nature and scale of the issue, figure 1 summarizes the most recent official Work Programme job outcomes data published by the DWP in December 2013, which covers the first 27 months of the programme's operation. This is, admittedly, early data in the lifetime of the scheme but it does suggest problems in the extent to which the current differentiated payments design is effectively calibrating provider incentives *between* payment groups. At its simplest level, the differential payments across the claimant groups should at least be calibrated so as to equalize providers' incentives to work with (of course only notional) ‘average claimants’ *between* the separate claimant groups as Devereux's evidence to the Public Accounts Committee, cited above, focuses on. *If* the differentiated payment system is effectively calibrating providers' incentives *between* the Work Programme's claimant groups in terms of some idea of the ‘average claimant’ within each of these groups, then one would, on average, expect the job outcome rates to be relatively evenly balanced between the various claimant groups. Figure 1, however, shows in contrast that there are considerable imbalances in job outcome rates between the claimant groups, suggesting underlying imbalances in the extent to which the current payment levels are equalizing the balance between costs, risks and returns across these claimant groups. Whilst the overall job outcomes rate comes out at just under 15 per cent (the horizontal line) two groups are doing markedly better than this average, and a number of payment groups are doing markedly less well.

Part of the hesitance of providers in adopting the DWP's claimant groups as their own framework for targeting claimants reflects their recognition that the groups are relatively crude and with significant internal heterogeneity. This is well known both by policymakers and within the academic literature yet the persistence of the differentiated payments model attached to such broad and internally diverse claimant groups has significant implications for claimants in terms of their increased exposure to systemic risks of creaming and parking. As a consequence, and in contrast to Robert Devereux's response to the Committee of Public Accounts, cited above, the appropriate question in terms of seeking to design out creaming and parking is not whether the current programme design is more subtle than previous welfare-to-work schemes but, rather, whether it is adequate to overcome creaming and parking. Although it remains early days for the Work Programme, the evidence accumulating here and elsewhere (Newton *et al*. 2012; Lane *et al*. 2013; PAC 2012, 2013; WPSC 2011, 2013; Rees *et al*. 2013) suggests not. The first phase of the DWP-commissioned qualitative evaluation of the Work Programme, for example, is surprisingly frank for an official evaluation: ‘the available evidence to date suggests that providers are engaging in creaming and parking, despite the differential payments regime’ (Newton *et al*. 2012: 124).

Cost pressures, combined with ambitious targets, were seen by many providers as a basic but central issue and there was a widespread perception that whatever process and design improvements might be made that the underlying reason for creaming, parking and poor performance against the DWP's minimum performance standards was under-resourcing of the programme. As one experienced operator in the welfare-to-work field commented:

*‘Regardless of what the government are saying … they haven't funded it properly to be able to get a good service. They wanted to move to an all encompassing service and they had an ideal opportunity to do that, and I for one thought it was brilliant that they did that … and they had a really good opportunity to make sure that that was funded properly so that we really could see improvement in people going back into work and it's just not happening, is it? Or it's not happening on the scale that they wanted it to’* (tier 2 provider)

Within this pressurized context, primes were acutely aware that claimant groups were a blunt instrument oriented primarily around the prior benefit received and not necessarily coterminous with a customer's distance from the labour market. This could work in either direction, with some individuals placed in relatively ‘job ready’ groups attracting relatively low potential payments (e.g. JSA 18–24 or JSA 25+) actually being perceived by providers to face serious barriers to moving into paid work. Conversely, some individuals placed in relatively ‘hard to place’ claimant groups and thus attracting substantial financial payments for sustained job outcomes (e.g. ESA ex-IB^4^ claimants) may actually be assessed by primes as needing relatively little support to move into paid work – the ideal client from a prime provider's perspective. This inevitably left providers reflecting on these frequent mismatches between their own evaluation of the individual's distance to the labour market and the Work Programme's evaluation as proxied by the level of financial payment attached to that individual's claimant group. As one third sector organization described, *‘We've got a guy who carries around a mirror in his pocket to ward off evil spirits. Okay he might be on JSA but he's a long way from the job market isn't he?’* (tier 1 provider). Another provider in a different CPA noted the extent of undiagnosed mental health issues amongst JSA claimants:

*‘There have been a lot of undiagnosed mental health conditions, as secondary [pause] as secondary illnesses to what's actually going on … some of these people have got extremely complex barriers before they even think about going into work … And yet they're a JSA customer and they [pause] the number of times that I want to say, ‘This person should not be on the Work Programme, they're probably a work [pause] if anything, they're a Work Choice*^5^ *customer … or they need to be left alone for at least six months and helped to sort out the other issues that they have.’* (tier 2 provider)

One interesting finding is that whilst the creaming and parking debate, both here and elsewhere, tends to be framed in the language of incentive structures and rational economic behaviour, there is evidence that some parking might arise inadvertently because of the inexperience or inadequate information held by providers and that there might be a learning curve to go through similar to that seen in the Job Network (Dockery and Stromback 2001). One end-to-end provider, for example, described how job advisers within a particular prime might lack the knowledge (and are bowed by pressure from high case loads) to refer jobseekers to appropriate sub-contractors, by implication leaving them to be parked. Additionally, the initial assessment is supposed to ‘flag’ customer needs but this was not, apparently, working effectively. They, therefore, decided to send their own staff to work alongside job advisers to ‘drive’ referrals to the sub-contractor:

*‘Our workers are backing [named prime] officers making sure that people remember to refer people to [named tier 1 provider], that actually if you've got somebody who's got a substance misuse or mental health issue you're better off referring them to [same tier 1 provider] than holding onto them and not being able to get them a job.’* (tier 1 provider)

Another provider commented similarly that whilst assessment tools may be widely used they are also far from comprehensively developed or utilized such that frontline advisers were sometimes ill-prepared to refer effectively:

*‘And actually, will those frontline advisors know what to do with that customer? Doubt it. Probably park them. But what I thought was, right, let me come up with something that takes the best of what Australia have got in terms of assessment, using health professionals, occupational therapists, develop that in the UK to make an assessment that's face to face, that actually gives direction for that customer so what happens is now [is progression to specific services].’* (tier 2 provider)

**Figure 2:**
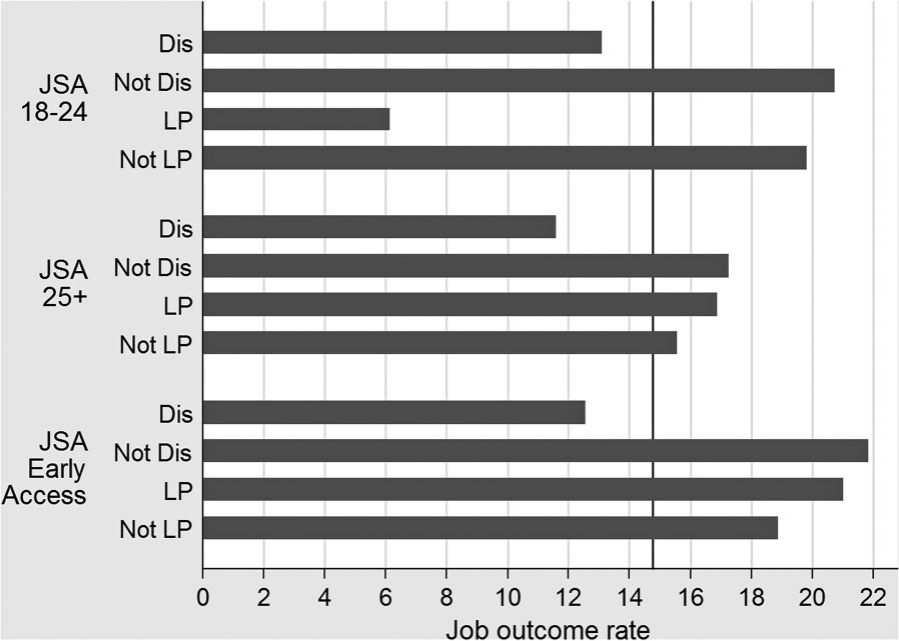
Differential job outcomes within Work Programme claimant groups

Whilst potentially emerging from informational as well as economic motivations, therefore, the quantitative material presented in figure 2 is also consistent with, and lends support to, the idea that the current Work Programme design does not adequately mitigate incentives to cream and park across different types of claimants *within* claimant groups in addition to simply *between* those groups. To explore this issue, figure 2 focuses on differences in job outcome rates between claimants with and without employment ‘disadvantages’ relating to disability and single parenthood across the three largest payment groups which together make up around 80 per cent of referrals to date. The rationale for these analyses is that single parents and the disabled might be expected to be ‘harder to place’ for providers and, as such, might rationally be expected to be more vulnerable to parking.

The trend in figure 2 for lower outcome rates amongst the relatively ‘disadvantaged’ payment group members is seen clearly and consistently across the three payment groups when comparing participants with a reported disability against those with no disability. Drawing on the social model of disability, where societal barriers operate to prevent disabled people from participating as equals in society (Barnes and Mercer 2003), there are additional barriers faced by disabled people seeking work (Patrick 2012), and the additional costs associated with overcoming these barriers may be at play in lower outcome rates for this group. Clearly, these data do not prove that creaming and parking are taking place, but they are in line with that practice and at a minimum highlight that the current differential payments structure is not calibrated to individual variation.

The chronic scarcity of care compatible employment opportunities (Gingerbread 2010) and lack of affordable local childcare (Daycare Trust 2012) stimulate an expectation that lone parents may face particular difficulties in securing sustained work from the Work Programme. Figure 2, however, shows that the story from the Work Programme so far is a little more complex in that whilst younger lone parents fare consistently less well than younger non-lone parents in terms of their job outcome rates older lone parents fare consistently better than older non-lone parents. It is unclear why these findings should be seen, although it might be partly due to the fact that younger lone parents have younger children (Coleman and Lanceley 2011) and that, for the reasons outlined above, younger children present stronger obstacles to lone parent employment (Bryson *et al*. 1997). Older lone parents also tend to have stronger human capital and work histories than younger lone parents (Coleman and Lanceley 2011).

The JSA Early Access group comprises three separate types of claimants: mandatory entry of 18-year-olds not in education, employment or training (NEETs); mandatory entry of JSA ‘repeaters’ (those receiving JSA for 22 of the past 24 months); and voluntary early entry for pre-identified ‘vulnerable’ JSA claimants (DWP 2013). Whilst it is impossible to say categorically from the publicly available data, it seems most likely that lone parents have been recruited to this group as JSA repeaters and thus to have some recent labour market experience. When compared with lone parents in the JSA 18–24 payment group, lone parents in the JSA Early Access group are also likely to be generally older and to have older children. Moreover, whilst job outcomes are triggered only after six months of sustained work for the JSA payment groups, this occurs after three months for the JSA Early Entry group, which will certainly help to make the job outcome rates in this group seem relatively more impressive than the other two JSA groups.

**Figure 3:**
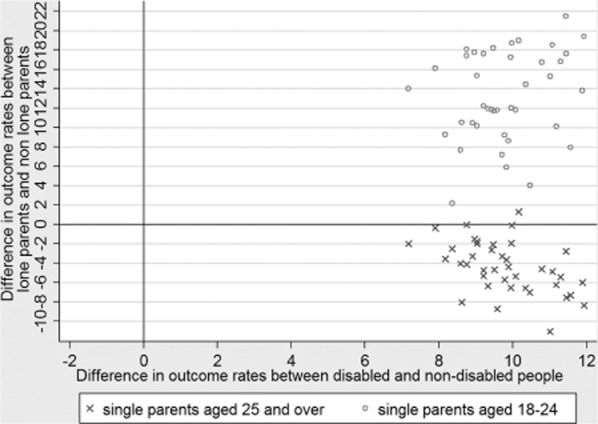
Patterned inequalities in job outcomes across Work Programme contracts

To explore these issues further our analyses take advantage of the fact that the Work Programme is broken down into 40 separate contracts with primes across the 18 CPAs, with some primes delivering across several CPAs and so having several contracts. Figure 3 extends the quantitative analyses by focusing on the question of consistency in results, again making use of the ability within the official data to disaggregate job outcomes by disability and single parenthood. For each Work Programme contract, figure 3 shows the difference between the disabled and non-disabled job outcome rates (horizontal axis) and the difference between the single parent and non-single parent job outcome rates (vertical axis). For both axes, a difference of zero implies identical job outcome rates between the two groups, positive values mean that the non-disabled/non-single parent job outcome rate is higher than the disabled/single parent job outcome rate, and negative values mean the opposite. Figure 3 is presented as a quadrant, and if the Work Programme's model of claimant groups and differential payments was successfully calibrating providers' incentives across claimants then one would expect any resulting differences in rate differences to be due to chance rather than anything systematic. In this case, the points would tend to centre around zero at the intersection of the two lines shown and to show a fairly random cloud of points around that intersection.

In contrast to this neutral picture, figure 3 highlights across the horizontal axis that disabled participants experience markedly lower job outcomes than non-disabled participants in every Work Programme contract. Looking along the vertical axis, there is also a consistent pattern in most (though not all) contracts that younger lone parents fare markedly less well than younger non-lone parents. In contrast, however, it is interesting to see that older lone parents tend to see somewhat better job outcome rates than older non-lone parents, the result perhaps of the combination of weaker needs for care-compatible work and childcare along with evidence showing that lone parents have a particularly strong motivation to work (Tu and Ginnis 2012). If differential job outcome rates between ‘easier to help’ and ‘harder to help’ claimants are accepted to be an indicator of potential parking then the consistency in these findings across the 40 contracts lends weight to the notion that strategies, practices and cultures of prime providers in relation to creaming and parking may well be involved.

## Conclusion

The Work Programme represents a radical extension of the incremental evolution of employment services witnessed in recent years in the UK. It implements for the first time, at a national level, a fully outsourced ‘black box’ model with payments based almost entirely on job outcome results. The programme aims to realize the key tenets underpinning international welfare-to-work reform over the past two decades but appears to be vulnerable to multiple tensions inherent within its design. This article focuses on the challenge for the all-encompassing Work Programme to calibrate the incentives for providers to work differently, but equally, in meeting the specific support needs of all jobseekers. The DWP sought to mitigate the identified risks around creaming and parking primarily through the placement of each individual into one of nine claimant groups, each with different levels of financial payments for job outcomes scaled according to a notion of the average difficulty of securing transitions to employment in each. The DWP's hope is that, if designed appropriately, differentiated payments across claimant groups would turn the rhetoric of ‘differentiated universalism’ into policy reality, mitigating providers' incentives to cream and park.

The combination of the quantitative and qualitative data presented above, however, suggests that this has not been achieved in practice and that creaming and parking may also be systemic in that they flow directly from the current design of the Work Programme. *If* the differentiated payment system was effectively calibrating providers' incentives *between* the Work Programme's claimant groups then one would, on average, expect the job outcomes rates to be relatively evenly balanced between the various claimant groups. This is not the case in practice, suggesting underlying imbalances in the extent to which the current payment levels are equalizing the balance between costs, risks and returns across these claimant groups. Disaggregating the data for ‘harder to place’ groups across the 40 Work Programme contracts displays an alarming degree of consistency in the findings that disabled people and young lone parents experience relatively lower job outcome rates than their ‘non-disadvantaged’ peers. Far from delivering ‘differentiated universalism’, the Work Programme at present seems instead to be reinforcing, exacerbating and making systemic the negative impacts of employment disadvantages.

Therefore, and in response to the question posed at the outset of the article, it is extremely difficult to argue on the basis of this evidence that the DWP has succeeded in designing the Work Programme payment groups and differential payments such that they mitigate providers' incentives to cream and park different individuals either across or within its broad payment groups, all in a context where providers are experiencing intense pressures around costs, cash flows and performance. Clearly, these quantitative data alone do not *prove* that parking is taking place – one needs to align the evidence on outcomes with the qualitative evidence around Work Programme processes for that – but the patterns seen are perfectly in line with what would be expected *if* parking were occurring. When taken together with the various emerging qualitative evidence discussed here, from the official Work Programme evaluation (Newton *et al*. 2012; Lane *et al*. 2013) and from government select committees (PAC 2012, 2013; WPSC 2011, 2013), then the notion that creaming and parking are serious problems within the Work Programme becomes compelling.

It will be of particular interest to an international audience that this evidence has been found despite the progression to what, at least superficially, appears a more complex and nuanced framework from the DWP in terms of payment groups and differential payments. Whilst value for money arguments from policymakers support a heavy weighting of payments onto job outcomes this strengthens the ever present challenge to mitigate providers' incentives to focus their energies and resources where it will pay. Value for money arguments such as these carry risks around parking, not just for claimants but also for ongoing social security budgets for those who fail to be supported into work. At present, it seems the Work Programme design may not have struck the right balance between value for money, incentives and claimant protections. With a challenging economic backdrop constraining job outcomes and with providers – and government select committees (WPSC 2013) – united in questioning the adequacy of resources within the programme to meet the support needs of more challenging claimants, such risks and weaknesses in the programme design are magnified. The challenge for UK and international policymakers seeking to embrace quasi-marketized welfare-to-work delivery is to drive forward the evolution of their programmes, such that they better balance their inevitable tensions between efficiency and equity.

## Notes

1 The North East, North West, Scotland and Wales are all examples of the scale of CPAs.

2 Authors' calculations based on Inclusion 2011b.

3 The RAG rating uses a traffic light system to group individuals either as red (hard to move into work), amber (moderately difficult to move into work) or green (easy to move into work).

4 Incapacity Benefit (IB) is currently being replaced by the Employment and Support Allowance (ESA).

5 Work Choice is a separate employment programme offering support to individuals with disabilities and long-term health conditions.
